# Palpable Penile Metastases: A Bizarre Presentation of Rectal Adenocarcinoma

**DOI:** 10.1155/2015/876464

**Published:** 2015-09-08

**Authors:** Liza Cholin, Sarah Perz, Furman Mahmood, Saleem Zafar

**Affiliations:** Department of Urology, University of Toledo Medical Center, 3000 Arlington Avenue, Mailstop 1091, Toledo, OH 43614, USA

## Abstract

Metastasis to the penis is an uncommon occurrence, with only about 370 cases reported in the literature to date. The majority of the primary tumors are genitourinary in origin. We report on a patient with undiagnosed disseminated rectal adenocarcinoma, who first presented with lesions of the corporal bodies. A review of the literature indicates that corporeal metastasis as an initial presentation of malignancy is an extremely rare occurrence and carries a very poor prognosis.

## 1. Introduction

Despite the penis being an organ with rich vascularization, metastasis to this site is an extremely rare event. The majority of cases involve primary tumors from the pelvic region including bladder (30–35%), prostate (28–30%), rectosigmoid colon (13%), kidneys (8–10%), and testes (5%) [1, 2]. Other less common primary sites include the gastrointestinal and respiratory tract. Regardless of the site of origin, the majority of cases (90%) have disseminated disease at the time of presentation [3] and thus are associated with a poor prognosis. One-third of penile metastases are diagnosed in synchrony with the primary tumor, while the remaining two-thirds are found within 18 months of diagnosing the primary tumor [4]. To the best of our knowledge, this case is the first reported with penile metastases as the initial presentation.

## 2. Case Report

An 88-year-old man presented to his urologist with a new complaint of penile lesion. The lesion was on the dorsum of the penis and midshaft and very tender. It appeared rather abruptly and was not associated with any recent trauma. He had urologic history including left orchiectomy and left groin radiation many years ago, benign prostatic hyperplasia (BPH) treated with transurethral resection of the prostate (TURP) twice, overactive bladder treated with oxybutynin, low testosterone treated with transdermal testosterone replacement, recurrent prostatitis, and irritative voiding symptoms. He had a history of prostate biopsy for elevated PSA and palpable nodule which was negative for malignancy as were all of his TURP specimens. He had also underwent urodynamic studies and multiple cystoscopies. Approximately 18 months prior to the appearance of the penile lesion the patient began complaining of intermittent pelvic pain, which usually resolved with treatment for prostatitis. His medical history also included hypertention, hyperlipidemia, arthritis, and chronic diarrhea.

**Figure 1:**
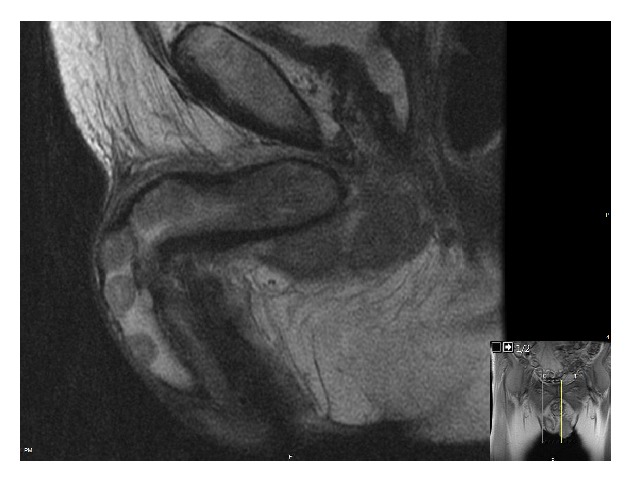
MRI showing low signal lesions invading the corpora cavernosa.

On physical exam the patient had hard nodules in the dorsum of his penis at the midshaft, which were exquisitely tender to palpation. These lesions were not clearly consistent with Peyronie's disease; therefore he underwent magnetic resonance imaging (MRI) of the pelvis which showed multiple nonenhancing low signal lesions in the corpus spongiosum and bilateral corpora cavernosa suggestive of metastatic disease (Figure 1).

**Figure 2:**
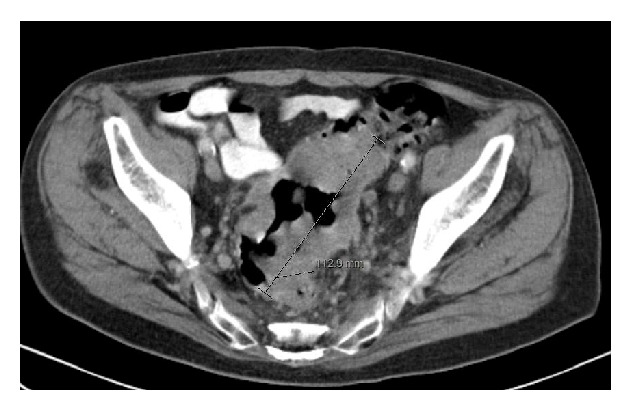
CT scan revealing 11.3 cm sigmoid mass.

An incisional biopsy was performed which confirmed adenocarcinoma with colonic origin. He next underwent a computed tomography (CT) to evaluate the extent of disease which showed 11.3 cm sigmoid mass with multiple enlarged lymph nodes adjacent to the sigmoid as well as in the retrocrural, para-aortic, and parailiac regions. It also demonstrated lesions consistent with metastases in the liver, adrenals, and lungs (Figure 2).

The patient was referred to hematology-oncology. His overall condition began to deteriorate rapidly including decreased exercise tolerance, decreased appetite, and weight loss. Because he would not likely tolerate systemic chemotherapy he was offered palliative treatment in the form of targeted chemotherapy after testing his tumor for genetic mutations. The patient requested time to consider his options. He passed away at home 5 weeks after his biopsy and 9 weeks after he initially presented with penile lesions.

## 3. Discussion

The first report to ever describe metastatic disease to the penis was in 1870 [5]. Colonic and rectal carcinomas are more likely to spread to other intra-abdominal organs, such as the liver. However, it is important for a clinician to consider metastases in cases of atypical penile lesions in order to avoid a missed or delayed diagnosis.

### 3.1. Clinical Presentation and Differential Diagnosis

Initially one should consider more common diseases which can cause penile or corporal lesions when developing a differential diagnosis for a patient complaining of penile lesions. Benign lesions of infectious (tuberculosis, syphilitic chancre, and chancroid) and noninfectious (benign priapism and Peyronie's disease) etiologies should be considered. Primary tumors of the penis are much more common than secondary tumors, and one should consider squamous cell carcinoma, invasive penile carcinoma, sarcoma, melanoma, and extramammary Paget's disease in the differential as well. In two case reports [6, 7], the patients' penile lesions were initially mistaken for Peyronie's disease. Thus, an atypical presentation of Peyronie's disease may warrant further investigation to rule out rare disease such as metastases.

Metastatic lesions to the penis or corporal bodies may present with a wide range of complaints. The most common presenting complaint is priapism (40%), followed by urinary retention, penile nodules, ulceration, perineal pain, edema, generalized swelling, broad infiltrative enlargement, dysuria, and hematuria [3]. Most often, the patient will have a penile mass. In over half of reported cases, the metastatic lesions initially appeared as infiltrative nodules, with most having bilateral involvement of the corpora cavernosa [1].

### 3.2. Routes of Metastasis

Several routes of metastasis to the penis have been proposed including retrograde venous spread, retrograde lymphatic spread, arterial spread, direct extension, and implantation secondary to instrumentation [1]. Retrograde venous route is the most common route [1, 2] and is attributed to the dorsal venous system of the penis being in direct communication with the venous plexuses of the pelvic viscera. Thus, when there is an obstruction proximally (from tumors arising in the prostate, bladder, or rectosigmoid), retrograde flow occurs, allowing spread of tumor cells to the penis. Retrograde lymphatic flow occurs in a similar manner. The lymphatics of the penis share a common route (through the iliac nodes) with portions of the bladder, prostate, and lower rectum. This route is theorized to be the primary path of spread to the penile skin [1]. Arterial spread is uncommon. It is thought to occur either as direct tumor extension into the neighboring arteries or by tumor embolization. Direct extension requires tumors to be in close proximity to the penis (usually the prostate or bladder). Implantation secondary to instrumentation is unlikely and largely debated.

### 3.3. Diagnosis, Treatment, and Prognosis

In order to make a diagnosis of the penile lesion, a biopsy or corporeal aspiration is needed. It is important to differentiate primary versus secondary tumors, since this will dictate treatment. After obtaining a biopsy for pathologic diagnosis noninvasive imaging can be performed using PET, CT scan, or MRI in order to evaluate the extent of disease.

It has been demonstrated in the literature that most patients with penile metastasis have widespread disease at the time of presentation and treatment is often oriented toward palliation. This usually includes a combination of chemotherapy and radiotherapy. Priapism can be treated with dorsal nerve block or penectomy, if the pain is refractory to less invasive measures. Penectomy can also be performed with intent of cure, in the rare instances when a patient presents with local disease. Such a case was reported on a patient with recurrent rectal carcinoma, who presented with a localized lesion in the corpus spongiosum [4]. Unfortunately, the disease is often quite advanced and there is an estimated 80% six-month mortality regardless of treatment [5].

Metastases to the penis are an uncommon occurrence. Most of the cases reported have synchronous primary malignancies present or primary tumors that were previously diagnosed. Our case was unusual, in which the penile mass was the initial presentation of a nonurologic primary malignancy. While these cases are rare, clinicians should remain aware that such presentations can occur and keep them in their differential diagnosis. Regardless of the site of primary tumor or treatment modality, early referral to hematology-oncology is paramount as the prognosis remains poor for these patients.
